# The concepts and origins of cell mortality

**DOI:** 10.1007/s40656-023-00581-8

**Published:** 2023-06-08

**Authors:** Pierre M. Durand, Grant Ramsey

**Affiliations:** 1 Department of Philosophy, Stellenbosch University, Stellenbosch, South Africa; 2 Institute of Philosophy, KU Leuven, Leuven, Belgium

**Keywords:** Mortality, Programmed cell death, Cell death concept, Cell death origin, Evolutionary concept

## Abstract

Organismal death is foundational to the evolution of life, and many biological concepts such as natural selection and life history strategy are so fashioned only because individuals are mortal. Organisms, irrespective of their organization, are composed of basic functional units—cells—and it is our understanding of cell death that lies at the heart of most general explanatory frameworks for organismal mortality. Cell death can be exogenous, arising from transmissible diseases, predation, or other misfortunes, but there are also endogenous forms of death that are sometimes the result of adaptive evolution. These endogenous forms of death—often labeled programmed cell death, PCD—originated in the earliest cells and are maintained across the tree of life. Here, we consider two problematic issues related to PCD (and cell mortality generally). First, we trace the original discoveries of cell death from the nineteenth century and place current conceptions of PCD in their historical context. Revisions of our understanding of PCD demand a reassessment of its origin. Our second aim is thus to structure the proposed origin explanations of PCD into coherent arguments. In our analysis we argue for the evolutionary concept of PCD and the viral defense-immunity hypothesis for the origin of PCD. We suggest that this framework offers a plausible account of PCD early in the history of life, and also provides an epistemic basis for the future development of a general evolutionary account of mortality.

## Introduction

An implicit assumption in biological inquiry is that an organism’s lifespan is finite (e.g., Cole, 1954). Whether the result of external or internal causes, all organisms, no matter how long they live, inevitably die. This death allows species to climb fitness peaks while evolving complex, adapted traits. Death, however, is not a passive bystander in the history of life. There are “different ways to die” (Jiménez et al., 2009) and how and when an organism dies has consequences for its own fitness as well as the fitness of its neighbors (Durand et al., 2011). In recognition of this, mortality has become at important field of inquiry in biology and philosophy (Huneman, 2023).

All organisms, irrespective of their organization (unicellular or multicellular, solitary or population-structured), comprise cells. An understanding of cell mortality is thus foundational to understanding organismal mortality. The frameworks used for classifying cell death have a long history. Some can even be traced back to before cells themselves were identified. Aristotle, for example, contrasted death by “extinction” and “exhaustion” in *Parva Naturalia* (467b “On Youth, Old Age, Life and Death, and Respiration”) and Bichat referred to “accidental” and “natural” death in his study of life and death mechanisms in his “Recherches physiologiques sur la vie et la mort” (Bichat, 1800). Such distinctions fundamentally concern whether death is caused by external events or arises internally. This external/internal contrast has persisted and is used by modern mechanistic biologists in studies of cell death, as well as evolutionary biologists and philosophers of biology, who tend to adopt broader terms like cell and organismal mortality. Aristotle and Bichat’s classifications are now commonly recast in terms of *exogenous* vs. *endogenous* mortality. Exogenous—“extinction” (Aristotle) or “accidental” (Bichat)—death refers to external causes. Endogenous—“exhaustion” (Aristotle) or “natural” (Bichat)—death results from metabolism, aging, and other self-limiting or self-destructive development or behaviors.

The early studies concerning the mechanisms of endogenous death generally focused on phenomena like senescence and aging (Grignolio & Franceschi, 2012; Lemoine, 2020). Later, once cell death attracted more attention among biologists, other kinds of endogenous death were identified. Examples range from the toxin-producing genes that induce death in prokaryotes to intricately regulated, complex molecular pathways in protists, fungi, plants, and animals. The genetic basis for some of these kinds of cell death was first identified in animal ontogeny. Along with these discoveries came a dedicated term, *programmed cell death* (PCD). While PCD was first identified among multicellular organisms, it was later discovered in the unicellular world. This discovery set off a flurry of debate about whether PCD truly occurs in unicells and how such PCD should be defined and identified. PCD in unicellular organisms is especially interesting to evolutionists and philosophers (Durand & Ramsey, 2018) because organism-level selection, as it is traditionally interpreted, works against the maintenance of traits that decrease fitness. PCD carries an absolute fitness cost to those that exhibit the trait, which makes it puzzling that PCD not only exists, but is widespread and has an evolutionary history potentially as long as cellular life itself (La et al., 2022).

In this article we focus on two unresolved issues in the history, philosophy, and biology of cell mortality, especially PCD. The first is how PCD should be conceptualized. Our analysis begins in Sect. 2 by tracing the history of cell death studies with an emphasis on the neglected discoveries of the nineteenth century. The concepts of cellular exhaustion, natural cell death, and (later) PCD have, sometimes unintentionally, been revised several times since the terminologies were introduced. We then proceed from this historical context to examine and evaluate current PCD concepts.

The second issue concerns the relationship between the original PCD concept and the explanations for the origin and evolution of PCD. In Sect. 3 we structure the arguments put forward for the evolution of PCD into coherent hypotheses. In Sect. 4 we analyze these hypotheses and argue that the evolutionary PCD concept is helpful in clarifying the origins hypotheses and in providing an epistemic basis for future studies of cell death and the development of an overarching evolutionary account of mortality.

## Concepts of cell mortality

Ways of conceptualizing “natural” or “programmed” death have a long history and no univocal account has emerged. In this section, we examine the history of cell death studies and the diversity of approaches to cell death that have risen from this history.

### The discovery of natural cell death

Biologists usually present the history of cell death studies as beginning with works from the early and middle twentieth century describing non-incidental cell death in animal embryos. However, as some historians of biology have uncovered, notably (Clarke & Clarke, 1996), there is a substantial body of work from the nineteenth century that contributed to our initial understanding of cell death. This work has been largely overlooked by modern researchers despite the similarities between nineteenth and twentieth century microanatomical descriptions of dying cells. It is also important to note the significance of the context in which the nineteenth century findings were reported. They had already tied mortality to the evolution of multicellularity and set the foundations for the first mechanistic conception of cell death. This early work may also have inadvertently influenced others to assume that unicellular organisms were immortal.

Clarke and Clarke suggest that the erroneous belief that the discovery of naturally occurring cell death didn’t occur until the twentieth century may stem from the fact that nineteenth-century embryologists appear to have had a “preconceived notion that ontogeny (and phylogeny) were progressive” (Clarke & Clarke, 1996, p. 81), which was clearly not the case in the metamorphic animals they studied. (Metamorphosis and ontogeny are distinct phenomena.) The assumption was made, therefore, that nineteenth-century embryologists could not have appreciated the non-incidental nature of cell death and the implications for future studies of organismal mortality. However, a close examination of the earliest descriptions of cell death reveals that eighteenth-century embryologists were keenly aware of the significance of what they called “natural” cell death, especially when reporting on non-metamorphic development. The descriptions, terms, and contexts of cell death studies from the nineteenth century undoubtedly influenced those that followed, and some biologists continue to tie the evolution of PCD to animal development and tissue homeostasis.

Reports of microanatomical structures in multicellular tissues that resemble dead cells can be traced to the early nineteenth century, but the subject only attracted focused interest once a “unified cell theory of life” was articulated in 1839 (Schleiden, 1839; Schwann, 1839). Perhaps the most influential of the earliest works arrived with the publication of Carl Christoph Vogt’s (Vogt, 1842) monograph, whose title is translated as “Investigations on the developmental history of the midwife toad (*Alytes obstetricans*).” He provided unambiguous evidence for the hypothesis (later proved correct) that organismal development was due to the “natural” destruction of cells and their subsequent replacement by new cells. The competing hypothesis that cells transform from one kind to another was thus refuted.

The analysis of Vogt’s monograph by Clarke and Clarke (1996) indicates that he never used the term ‘cell death’ in his study of the anuran notochord and surrounding cartilage. The German equivalent, ‘zelltod’, emerged later in the nineteenth century. Vogt’s observations are usually referred to as “cell destruction” because he describes cells disappearing, being resorbed or destroyed. His descriptions provided the crucial clue that cells were dying naturally as opposed to their death being induced by external damage. His usage of the term ‘natural’ was synonymous with Bichat’s French terminology for non-accidental death. It was a prelude to the later conceptualizations of endogenous and programmed cell death, and it can be argued that his original terminology was equally appropriate. Vogt did not pursue his interest in animal development and moved into philosophy and politics, where he is unfortunately known for his untenable views on race. Nevertheless, by the middle nineteenth century the importance of natural cell death for development in multicellular organisms was already cemented (Kaczanowski, 2016).

While a comprehensive history of cell death research is beyond the scope of this paper, it is nevertheless important to emphasize that additional significant advances were made in the middle and late nineteenth century. There were contributions by Prévost and Lebert (1884), Weismann (1863), and many others (e.g., Stieda, 1872). (A more expansive list can be found in Table 1 in Clarke & Clarke, 1996). Insects and anurans were the dominant model organisms, but mammalian tissues also became a focus (Weismann, 1863). During this period many new cell death terms were introduced to describe a range of morphological features and mechanisms. These include histolysis, necrosis, necrobiosis, fatty degeneration (Virchow, 1860), autophagocytosis (Margo, 1862), and chromatolysis (Flemming, 1885). We mention these specifically because the same morphotypes were “rediscovered” in the twentieth century and given different names. ‘Necrobiosis’ became ‘shrinkage necrosis’ then ‘apoptosis’, but a review of the earlier nineteenth century reports indicates that the cellular morphologies were essentially the same. The contextual association between cell death and the ontogenetic fate of animals was highly significant from the beginning and continued for both historical and technical reasons (such as the methodologies being better suited to animal tissues), persisting for more than 150 years (Clarke & Clarke, 1996). Even now that the origin and evolution of cell death have been revised, many researchers cling to a concept of PCD and its origins that is inextricably tied to the ontogenetic fate of macroscopic organisms (Huettenbrenner et al., 2003).

In the late nineteenth and early twentieth centuries, natural cell death research continued apace in Germany and France resulting in dozens of articles (e.g., Collin, 1906; Conheim, 1889; Janet, 1907; Kallius, 1931; Pérez, 1910; Terre, 1889). The studies of cell death in vertebrates by Ernst and Mühlmann (Ernst, 1926) are worth highlighting. Ernst, in particular, produced voluminous analyses of ontogenetic cell death in hundreds of serial sections and dozens of species. He referred explicitly to the endogenous and exogenous factors, which played an important role in maintaining the distinctions originally introduced by Aristotle and Bichat. By the early twentieth century, the different kinds of natural cell death essential for animal development had become a mainstream interest (Hamburger, 1992). The full range of morphological features that characterized the different kinds of cell death was being catalogued and, in 1951, Glücksmann published an extensive list of the kinds of endogenous cell death found in vertebrate ontogenesis (Glücksmann, 1951; Flemming, 1885) and others (e.g., Beard, 1889) had already introduced terms describing nuclear and chromatin changes (e.g., chromatolysis) and Glücksmann drew further attention to the nuclear hallmarks of cell death such as “karyorrhexis” (nuclear fragmentation) and “karyopyknosis” (nuclear condensation), which are still used routinely. These descriptions and interpretations were important for the future development of—and philosophical reflections on—PCD concepts.

### The mechanistic concept of programmed cell death

In the second half of the twentieth century, it became clear that natural cell death followed a series of regulated steps, which Saunders described as a “death clock ticking” (Saunders, 1966, p. 606). The inexorable march toward death seemed to pass through ordered stages, prompting the coinage of the “programmed cell death” (PCD) label (Lockshin & Williams, 1964). The invocation of a program appeared highly appropriate for biologists, especially once the causal genes (Yuan & Horvitz, 1990) and cognate proteins (Lockshin, 1969) responsible for cell death morphotypes were being uncovered. In a time when molecular biology was flourishing, the term had great appeal and quickly became entrenched to describe endogenous cell death in animals, plants, and facultatively multicellular eukaryotes like slime molds.

At this stage in the science of endogenous death, there were two assumptions at the heart of how PCD was conceptualized. The first was that, as the name implied, PCD involved a biological program, which was the basis for the original gene-based mechanistic concept (see Table 1). An example—taken from the unicellular literature but a good reflection of the mechanistic conception of PCD in multicellular organisms—is the Berman-Frank definition of PCD as “active, genetically controlled, cellular self-destruction driven by a series of complex biochemical events and specialized cellular machinery” (Berman-Frank et al., 2004, p. 997). However, the continued use of ‘program’ in cell death has rightly been questioned (Ratel et al., 2001). PCD can be inherited epigenetically (Leung et al., 2022) and exhibits a high degree of phenotypic plasticity (Zeballos et al., 2023) and there is, of course, the more general philosophical issue concerning the nature of a program and whether it is justified to say that death is in these instances programmed. The second assumption, which we will return to in Sect. 3, concerns the origin of PCD. The mechanistic PCD concept was placed firmly in the context of tissue homeostasis and since unicellular organisms do not have multicellular tissues, the expectation was that the phenomenon was a hallmark of multicellular evolution.

**Table 1:** Cell death concepts. Programmed cell death (PCD) concepts stemming from different disciplinary approaches

Concept	Definition of PCD	Key References
**Mechanistic**	“Active, genetically controlled, cellular self-destruction driven by a series of complex biochemical events and specialized cellular machinery.”	(Berman-Frank et al., 2004, p. 997)
**Evolutionary**	Adaptive cell death. (This contrasts with ersatz PCD or non-adaptive cell death.)	(Durand & Ramsey, 2018)
**Developmental**	A developmental stage in the lifecycle of a unicellular organism.	(Cornillon et al., 1994; Hosoya et al., 2007; Wireman & Dworkin, 1977)
**Ecological**	A stress response and a mechanism for nutrient recycling within and between trophic levels.	(Franklin et al., 2007)
**Immunological**	A cell death-inducing immune response.	(Berngruber et al., 2013; Débarre et al., 2012; Fukuyo et al., 2012; Koonin & Zhang, 2017; Refardt et al., 2013)

### Elaborations on programmed cell death: apoptosis and other cell death morphotypes

Very soon after the adoption of the mechanistic PCD concept, specific names started being allocated to microscopically distinct cell death morphotypes. The morphologies (whose distinct genetic underpinnings were discovered later) were given names such as apoptosis, autophagy, necroptosis, oncosis, cornification, ferroptosis, and others to capture unique cellular or biochemical features. To ameliorate the confusion that subsequently emerged, groups of prominent researchers recommended a standardized nomenclature for cell death in multicellular organisms (Kroemer et al., 2005), and more recently for unicellular yeast (Carmona-Gutierrez et al., 2018). These have largely been adopted, although some astute researchers raised valid criticisms concerning the historical accuracy of the cell death nomenclature as well as the conflation of names and their meanings. Apoptosis is a good example of how the terminologies, their historical meanings and implications, are sometimes confused.

Apoptosis is one of the most intensively investigated morphotypes of PCD, largely because of its role in tumorigenesis and animal development. The term was introduced into the medical literature by Kerr and colleagues (Kerr et al., 1972) who originally called it “shrinkage necrosis,” but later suggested the word apoptosis to capture “a basic biological phenomenon with wide-ranging implications in tissue kinetics” (ibid., p. 239). While apoptosis is mechanistically and morphotypically distinct, the aim of introducing the term was to capture and highlight the kinetics of cell death in tissue homeostasis. The histological significance of apoptosis for tissues is quite different from, say, autophagy or one of the other morphotypes of PCD. Labeling cell death as apoptosis, therefore, has implications for both the expected cell morphotype with its underlying molecular machinery *and* the tissues in which the dying cells find themselves.

While apoptosis is commonly conflated with PCD, “‘apoptosis’ should not be considered as a synonym of the terms ‘programmed cell death’, ‘cell suicide’ or ‘self-destruction’’” (Ameisen, 2002, p. 368). This conflation can be found within prestigious national and governmental medical information websites. Even the originators of the PCD label don’t hold that apoptosis should be distinguished from PCD. A book chapter coauthored by one of the originators says that “both terms are used in an essentially interchangeable fashion, and to insist like a scholastic on the purity of the terms no longer makes sense” (Zakeri & Lockshin, 2008, p. 3). The distinction, however, remains important because of the implications it has for tissue homeostasis. It is also significant for evolutionary biologists and philosophers of biology because apoptosis and PCD are associated with different evolutionary histories (Durand & Ramsey, 2018; Hanna & Abouheif, 2023).

In addition to the mechanistic and evolutionary implications concerning the usage of apoptosis, its introduction and significance has been scrutinized by historians of biology. The word was proposed because of the etymological meaning of *dropping off* or *falling off* such as occurs in autumnal leaves, and Kerr and colleagues wished to capture the importance of the kinetics between living and dying cells in their tissue environments. The term apoptosis, however, was not new. Esposti (1998) and later Diamantis et al. (2008) drew attention to its usage as far back as Hippocrates of Cos (ca. 460 − 370 BC), who introduced the original Greek ‘αποπτωσισ’ to describe the “dropping off” of dead tissues from gangrenous limbs. Kühlewein (1902) reported its usage in Hippocrates’s treatise MOXΛΙKON (Chap. 35). Apoptosis was also translated into Spanish and gained usage by 1878 (Fernandez-Flores et al., 2002) before its incorporation into English. Historians have suggested that the naming of the apoptosis morphotype reflects the neglect of earlier descriptions of cell death by nineteenth-century embryologists. Clarke and Clarke (1996) report that natural cell death morphologies equivalent to apoptosis were described in the late nineteenth century and already had names such as ‘chromatolysis’ (Flemming, 1885) or ‘necrobiosis’ (Virchow, 1860). Kerr et al. (1972) were likely unaware of these earlier uses, as evidenced by a footnote in their highly cited paper in which they credit a professor in the Greek Department at the University of Aberdeen for introducing them to the word.

### Programmed cell death in the microbial world

In the late twentieth century, PCD—or something like it, because investigators were not yet convinced that it truly was PCD—was identified in the unicellular world (Ameisen, 2002). Many of the biochemical markers and morphological features of PCD were demonstrated in microbes but using the ‘PCD’ term to describe death in single-celled life came under intense scrutiny (e.g., Nedelcu et al., 2011; Ratel et al., 2001). The primary criticism was that the original conceptualization of PCD could only be applied to multicellular contexts. The first issue raised was technical. Critics of unicellular eukaryote PCD rightly claimed that the methodologies used in multicellular tissues may be unsuitable for unicellular organisms because of differences in cellular architectures, metabolic activities, and so on. (For a recent review of methodological inconsistencies see Barreto Filho et al., 2023). The validity and interpretation of the empirical data were questioned. Some researchers suggested that there is unlikely to be PCD, as conceptualized by Lockshin and Williams (1964), in some protist lineages, claiming that the “dedicated molecular machinery required for the initiation and execution of regulated cell death has yet to be convincingly identified” (Proto et al., 2013, p. 58). The second criticism related to the concept of PCD itself and its relationship with organismal mortality. If PCD was exclusive to tissue homeostasis in multicellularity, then it could not be part of the unicellular world. Unicellular organisms may succumb to exogenous causes of death, but the assumption that they were potentially immortal seemed reasonable to most biologists and using a term like ‘program’, which implied a deterministic outcome, was misleading.

The evolutionary significance of unicellular PCD presented the gravest problem. In multicellularity, its evolution was easily justified by the prevailing theory that PCD originated to accommodate multicellular lifestyles. However, the growing evidence for PCD-like phenomena in unicellular eukaryotes still required explanation, so alternative concepts like ‘cell death programme’ (Ratel et al., 2001) or ‘active cell death’ (Nedelcu et al., 2011) were proposed to distinguish them from multicellular PCD.

Attempts to dislodge the PCD terminology in microbes have thus far failed—and for good reasons. First, the evidence is overwhelming that unicellular organisms (especially eukaryotes) do display some of the characteristic ultrastructural features of animal PCD and its many morphotypes (Kaczanowski, 2016; Moharikar et al., 2006) even if there are also significant differences resulting from the presence of organelles like chloroplasts in microalgae. Terms like “apoptotic-like” have sometimes been used to tread a middle line (Moharikar et al., 2006). Second, many of the homologs and protein domains (the “domains of death”) responsible for PCD in multicellular organisms were identified in both prokaryote and eukaryote unicells (Aravind et al., 1999). There remain conflicting interpretations about whether the homologous domains are also functionally homologous. They may be involved in life-sustaining functions in unicellular organisms and subsequently co-opted in early multicellular organisms for the evolution of PCD (Nedelcu, 2009; Koonin & Aravind, 2002). Nevertheless, the combination of the shared PCD morphotypes, and the common ancestry and overlapping biochemical activities of at least some death-related proteins convinced most researchers that the evolution of PCD, whatever its meaning in the philosophy of cell mortality, preceded multicellularity. There were additional justifications for staying with the PCD label in the unicellular world. The highly significant role of PCD-like cell death in microbial ecology lent further support to the argument that there was an evolved functional significance to unicellular PCD (e.g., Allocati et al., 2015; Franklin et al., 2007; Ndhlovu et al., 2021; Kojic & Milisavljevic, 2020). And the intensely dissected molecular mechanisms in prokaryotes revealed multiple simple, unambiguous, genetic programs dedicated to cell death.

The initial focus following the discovery of PCD in unicellular organisms was on understanding the ancestry of cell death mechanisms and shared morphotypes. There was steady progress in this area, but the questions concerning the historical, ecological, and evolutionary significance of PCD, and its contribution to the philosophy of the biology of mortality, proved more problematic. The mechanistic concept of PCD itself was of little value to ecologists and evolutionists and soon became an ill-defined term. PCD eventually become so vague that investigators sometimes found themselves in the perplexing situation of not knowing what they were studying. Biologists were struggling to make “sense of a paradox” (Bayles, 2014, p. 63), still asking the fundamental question, “programmed cell death: what is this?” (Ramisetty et al., 2015, p. 89). If the PCD terminology was to persist, a conceptual realignment of its meaning across unicellular and multicellular worlds and across disciplines became an imperative.

What transpired in the cell death field in the late twentieth and early twenty-first centuries demonstrates some interesting parallels with other conceptual issues in the history of biology, such as the species problem. A single, universally applicable species concept has been unattainable. Instead, a heterogeneity of species concepts have been proposed and adopted by specific disciplines (Hey, 2001). This trajectory of multiple discipline-specific concepts resonates with the revisions of PCD that subsequently emerged.

### The evolutionary concept

An evolution-based PCD concept was proposed to address the impasse in the interpretations of PCD in the unicellular and multicellular domains (Durand & Ramsey, 2018). In the unicellular literature, adaptive and non-adaptive arguments for PCD were typically presented as mutually exclusive hypotheses, although a closer examination revealed that endogenous cell death may be both depending on ecological context (Barreto Filho et al., 2023; Durand & Ramsey, 2018). For example, PCD may be maintained by kin/group selection in one lineage, while in another it is non-adaptive. In the first instance, it may be a population or group-level adaptive stress response (e.g., Kojic & Milisavljevic 2020; Vardi et al., 2007; Yordanova et al., 2022; Zeballos et al., 2023; Barreto Filho et al., 2022) or a mechanism for limiting viral infections (Refardt et al., 2013; Fukuyo et al., 2012; Koonin & Krupovic, 2019; Vostinar et al., 2019). In the second, it may be a side effect of starvation or another environmental insult (Nedelcu et al., 2011; Segovia et al., 2003; Kourtis & Tavernarakis, 2009; Kajikawa & Fukuzawa, 2020; Affenzeller et al., 2009).

Two distinct evolutionary strategies (both resulting in PCD) for dealing with environmental challenges were evident (Durand & Ramsey, 2018). In one case, it was an adaptation in both unicellular and multicellular organisms. In the second, it was an adaptation in animals and plants and a non-adaptive side effect in microbes. To harmonize our understanding of cell death, it was proposed that the distinction between evolutionary histories be captured by defining PCD more narrowly as an adaptation across all scales of life (Durand & Ramsey, 2018). Cases of apparent PCD not based on adaptations were ersatz. The justification for this distinction demanded that at least one piece of incontrovertible evidence for adaptive death be identified. Several emerged, especially in bacteria (Lewis, 2000; Bayles, 2014), with perhaps one of the most elegant experiments using *Escherichia coli*. Refardt et al. (2013) included all the necessary components of the mechanistic PCD concept in one experimental design and demonstrated that (a) death can be encoded (programmed) by a single genetic locus, which (b) is causally linked to the cell death trait, a trait that (c) prevailed in a group selection competition experiment.

### The developmental, ecological, and immunological concepts

In addition to the mechanistic and evolutionary PCD frameworks, PCD in the unicellular world has also been studied in developmental, ecological, and immunological terms. Development-focused studies have included diverse prokaryotes and unicellular eukaryotes. PCD is widespread in prokaryote lifecycles (Bayles, 2014; Lewis, 2000) and its role has been established in some model organisms like the gram-positive bacterium *Bacillus subtilis*. In times of environmental stress, a single *B. subtilis* cell produces an endogenous spore, which is subsequently liberated by lysis of the mother cell. This lysis occurs via PCD and the event involves a transition from vegetative cell to spore (Hosoya et al., 2007). Sporulation ensures a more durable stage of the organism’s lifecycle and once conditions improve, the spore germinates into a metabolically active cell, which can once again grow and reproduce vegetatively.

PCD has also been described in the lifecycles of social bacteria and amoebae, which exhibit features of simple, facultative multicellularity. During times of starvation, *Myxococcus xanthus* cells aggregate and differentiate into spores or vegetative cells. The majority of cells in the group (~ 80%), however, undergo PCD (Wireman & Dworkin, 1977) and the nutrients liberated by their corpses sustain the survivors during the period of resource limitation. Similarly, PCD is the basis for the cooperative behavior observed in the developmental aggregative stage of *Dictyostelium* amoebae. In the lifecycle of this organism, some cells die by PCD and form a stalk, which props up the differentiated spores in a fruiting body (Cornillon et al., 1994).

In the three examples above, the authors do not explicitly suggest a developmental definition of PCD, although they use terms like ‘lifecycles’ or ‘developmental program’ in their explanations. Nevertheless, the developmental framework differs from the original mechanistic one discussed earlier (Table 1). In both conceptions, investigators discuss cell death in terms of molecular mechanisms, but for developmental biologists, the reference point is a specific stage in the organism’s lifecycle. In the case of *B. subtilis*, the lifecycle comprises vegetative, sporulation, and germination stages. For mechanistic cell biologists, PCD can be a feature of any of these stages, but for developmental biologists, cell death is associated with sporulation. In *M. xanthus* and *Dictyostelium*, lifecycles comprise free living and aggregative forms and there is a similar distinction between PCD mechanisms and development. The developmental concept is reserved for the aggregative stages.

In the ecological literature, PCD is always related, in one way or another, to processes like primary production, energy budgets, and nutrient flow through food webs, or to trophic levels like populations, communities, and ecosystems. Such processes and organizational levels are at the core of ecological inquiry (Sutherland et al., 2013) and are the reference points for cell death studies. Examples include the role of PCD in the flow of nutrients in hypersaline and marine ecosystems (Bidle, 2015; Orellana et al., 2013) or in population/community dynamics (Franklin et al., 2007; Ndhlovu et al., 2021; Kojic & Milisavljevic, 2020; Zeballos et al., 2023). Again, ecologists have not formally proposed a PCD concept. However, as expected, the PCD paradox is discussed in terms that are specific to the discipline.

In the immunological framework, PCD is associated with prokaryote immunity. Some of the mechanisms associated with this immunity may have emerged very early in the evolution of life (Makarova et al., 2013). Immunity genes in both bacteria and archaea typically colocalize with PCD modules in defense islands and their functional coupling has recently received greater interest (Gao et al., 2022). The association between viral invasion and immune responses like cell death or dormancy, which is sometimes considered part of the PCD spectrum (Durand & Ramsey, 2018), has been demonstrated numerous times independently in bacteria, archaea, and unicellular eukaryotes (Bautista et al., 2015; Iranzo et al., 2015; Koonin et al., 2017; Koonin & Zhang, 2017; Pelusi et al., 2021).

The concept of PCD as a form of cellular immunity was proposed more formally this century (Benler & Koonin, 2020; Iranzo et al., 2015; Koonin & Zhang, 2017; Lacey & Miao, 2020) with the intention of capturing a range of endogenous cell death mechanisms across the unicellular world. It includes prokaryotes and eukaryotes and spans an evolutionary timeframe from the emergence of proto-cellular life to self/non-self-recognition in eukaryogenesis and multicellularity (Blackstone & Green, 1999; Iranzo et al., 2015; Michod & Nedelcu, 2004).

### A paradigm shift in the philosophy of cell mortality?

By the early twenty-first century, PCD was already confirmed in many prokaryotes and in at least nine species of phylogenetically diverse unicellular eukaryote taxa. This prompted immunologist Ameisen, in a heavily cited review, to suggest that a paradigm shift has occurred in studies of mortality (Ameisen, 2002). At the heart of the conceptual discontinuity in Ameisen’s paradigm shift is the rejection of the hypotheses that PCD (1) is an exclusive feature of multicellular tissues, (2) co-evolved with multicellularity, and (3) comprises a tightly regulated genetic program. The thinking that “regulated cell suicide would have been obligatorily counter-selected in single-celled organisms” (Ameisen, 2002, p. 372) was refuted, resulting in what we refer to as the ‘PCD paradox’.

Whether or not there has been a paradigm shift (in the Kuhnian 1962 sense) in either cell death studies or mortality research is debatable. Nevertheless, the understanding of PCD has undergone a substantial transformation. As we discuss below, most researchers agree that the exclusive relationship between PCD and the multicellularity-cooperation origin hypothesis is untenable. A dwindling number may still challenge this, but the conflict is now based more on concepts and interpretations than on empirical data (Pandey et al., 2018). Following Ameisen’s review (2002), the number of reports of PCD in microbes increased dramatically and now includes all major bacterial and unicellular eukaryote lineages. Archaea are the remaining domain in which PCD studies have been limited, perhaps because of the technical difficulties in culturing archaea.

The transformations of our understanding of cell death, especially PCD, initiated a search for a deeper understanding of mortality from both scientific and philosophical approaches. Huneman’s (2023) monograph *Death: Perspectives from the Philosophy of Biology* is an example of recent philosophical attention to these issues. This has emerged largely because of the uncoupling of mortality from multicellular organisms—unicellular life was for a long time considered immortal—and has prompted biologists to search for a clearer understanding of the origins of cell death.

## Hypotheses for the origin and maintenance of programmed cell death

Most researchers accept that PCD, however we wish to conceptualize it, arose in prokaryotes (eubacteria and archaea) or their common ancestor(s) (Koonin et al., 2017; La et al., 2022). The mechanisms and selective pressures driving cell death evolution in the earliest cells are unknown, although several ideas, hypotheses, and arguments have been proposed. In this section, we organize these proposals into five hypotheses (see Table 2). There is overlap between them, which we will highlight, and some authors suggest that different processes may have acted synergistically or occurred in parallel. At the same time, however, each hypothesis is unique and explains examples or data that cannot be accommodated by any of the others. One complication in assessing these hypotheses, however, is that in most instances, the distinction between the origin and maintenance of PCD is not made by their proponents. Origin and maintenance are, of course, different and we draw attention to this distinction in our analyses.

**Table 2:** Five hypotheses for the origin and/or evolution of PCD.

Hypothesis	Description	Key references
**Multicellularity and cooperation**	Adaptive cell death emerged with multicellularity	(Huettenbrenner et al., 2003; Nedelcu et al., 2011; Pandey et al., 2018; Segovia et al., 2003)
**Addiction**	Cells are unable to purge themselves of autonomous toxin genes	(Ameisen, 2002; Engelberg-Kulka & Glaser, 1999; Yarmolinsky, 1995)
**Pleiotropy and the original sin**	1. Life-sustaining activities like metabolism have harmful biochemical side-effects. 2. Life-promoting genes are pleiotropically linked to death-inducing mechanisms	(Ameisen, 2002; Nedelcu et al., 2011)
**Conflict mediation and coevolution**	1. PCD mediates group-level conflict. 2. PCD-based mechanisms coevolved with group level traits	(Blackstone, 2013; Durand, 2021; Michod & Nedelcu, 2004)
**Viral defense and immunity**	PCD emerged as an immunological defense against viruses	(Benler & Koonin, 2020; Berngruber et al., 2013; Fukuyo et al., 2012; Koonin & Krupovic, 2019; Vostinar et al., 2019)

### The multicellularity-cooperation hypothesis

The prevailing hypothesis has long been that PCD originated as a mechanism for cooperation between cells in multicellular organisms (Lockshin & Williams, 1964; Zakeri & Lockshin, 2008). The thesis was that PCD was a regulator of tissue dynamics (via ontogenesis and immunity against neoplasia), thereby aligning the evolutionary interests of individual cells in the multicellular organism. The multicellularity-cooperation hypothesis was subsequently extended to include social unicellular organisms that form aggregates, such as *Myxococcus* and *Dictyostelium*, which we discussed in the developmental concept (Sect. 2.6). Some traditionalists suggest that biologists should hold steadfastly to the multicellularity-cooperation hypothesis and that the PCD concept should be used only in the multicellular context and in social unicellular organisms. The argument is that this is how PCD was originally conceptualized—as a mechanism of cell-level cooperation in animals and plants. However, there is now a growing consensus that unicellular PCD is widespread and adaptive at multiple levels of organization. PCD has therefore become uncoupled from the evolution of multicellularity, which has cast doubt on the hypothesis that PCD arose solely in the context of multicellular organisms.

### The addiction hypothesis

The inability of prokaryotes to purge themselves of death-inducing autonomous DNA molecules was recognized in the middle to late twentieth century, leading to the term ‘addiction molecules’ (Engelberg-Kulka & Glaser, 1999; Yarmolinsky, 1995). Typical examples of cellular addiction to death are the plasmid-encoded toxins and their antitoxins, which function as antidotes to the death-inducing toxins (Jensen & Gerdes, 1995). Both toxin and antitoxin genes (TA modules) are obligately expressed and cells that inactivate or purge themselves of the plasmid or other extrachromosomal DNA molecules that harbor TA modules are left with stable toxins and labile antitoxin proteins in their cytoplasm. The stable toxins are longer lasting and, once the antitoxins have degraded, puncture holes in the cell wall leading to the death of the host cell. Plasmids and other autonomous genetic elements that encode TA modules invade and persist in their prokaryote hosts, because only cells that harbor functional TA modules produce antitoxins, survive, and reproduce. Cells become addicted to the TA modules and those that do manage to cure themselves of the parasitic elements are destined to die.

In proposing an origin for PCD, Ameisen formalized the argument as the addiction hypothesis (Ameisen, 2002, 2005), and pointed to its strengths: First, it is mechanistically simple and only a handful of genes are required. Second, it remains a common cell death mechanism in extant prokaryotes and is an example of a system that is both necessary and sufficient as a cause of PCD. The plasmids or extrachromosomal DNA molecules encoding TA modules are typically autonomous in that they are structurally and functionally distinct from the host cell genome. Over time the host cell may domesticate the TA modules and use them to poison competing cells, but Ameisen’s claim was that the origin of PCD was a gene-level adaptation.

### Pleiotropy and the original sin hypothesis

The pleiotropy-original sin hypothesis (POSH) is favored by those who argue that PCD is a non-adaptive side-effect of life-promoting activities. Ameisen (2002) made the point that life-sustaining processes like metabolism or reproduction produce harmful wastes and toxic by-products (e.g., oxygen free radicals), that can potentially cause death if the biochemical processes are not tightly regulated. The ‘original sin’ is that the evolution of life is unavoidably associated with biochemical reactions that promote death. “All cellular processes have intrinsic error rates,” Ameisen notes, and “most, if not all,. . .enzymatic activities that, if not tightly regulated, have the intrinsic potential to lead to cell death” (Ameisen, 2002, p. 385).

PCD can also be a pleiotropic phenomenon in which death is encoded by the genes themselves and is not simply a by-product of biochemical reactions. To illustrate this, Ameisen formulated a thought experiment, drawing on the example of the dual effects of autolysins discovered in some bacterial taxa. Autolysins digest the peptidoglycan bacterial wall during cell division, but they can also induce self-destruction when they are occasionally dysregulated in adverse environmental conditions. Similar multifunctional genes control the cell cycle, differentiation, and sporulation and participate, at various levels, in the regulation of PCD. PCD-related genes are both vital and dangerous and it is the cellular environment and ecological context in which they are expressed that determines whether the genes are pro-survival or pro-death. Proponents of the POSH suggest that there is no “bona fide genetic death program in cells” (Ameisen, 2002, p. 386). Instead, PCD genes are selected because their primary functions are pro-survival. Death is simply an unwelcome side effect in some environmental contexts.

The POSH has received some preliminary empirical support from molecular biologists and genomicists. Some of the effectors of PCD are indeed pleiotropically connected to non-death functions (Lee et al., 2008). In addition, recent comparative genomic data reveal that PCD domains may trace back to the earliest cells and are usually embedded in genes or regulatory regions with pro-life functions (La et al., 2022). These findings that suggest an ancient origin for PCD and a pleiotropic relationship with pro-life genes lend support to the POSH, although further corroborating data are required. There are, however, valid criticisms of the POSH. For example, the claims made by proponents of the hypothesis and most of the empirical support they cite comes largely from unicellular eukaryotes but the pleiotropic argument they make for the origin of PCD is relevant to the earliest cells, which were, of course, prokaryotes or more elementary proto-cellular organisms. The genomic architectures of prokaryotes and eukaryotes are also markedly different, and it seems overly ambitious to extrapolate the eukaryote genetic data to the earliest prokaryotes.

### Conflict mediation and the coevolution hypothesis

We have discussed two hypotheses for the adaptive evolution of PCD. The first is the argument that PCD may have originated as a gene-level adaptation in early prokaryotes (the addiction hypothesis) and the second is that PCD arose with the evolution of multicellularity (the multicellular-cooperation hypothesis.) However, adaptive PCD plays a much more general role in microbial sociobiology. Huneman suggests that “death is a social issue” (Huneman, 2023, p. 461) and, indeed, a range of unicellular interactions depend on cell death or use death as a mechanism of social communication. Eukaryogenesis is one of the prime examples. Proto-eukaryotes arose from the social interactions between unrelated prokaryotes that eventually gave rise to mitochondrial, plastid, and nuclear genomes. Theoretical arguments and mathematical models conclude that there was significant conflict between the cells giving rise to FECA, the first eukaryote common ancestor (Blackstone, 2013; Michod & Nedelcu, 2004). (This is still reflected in the genomic conflict observed in extant eukaryote cells (Burt et al., 2009).) However, for stable eukaryotes to emerge, conflict mediation was essential and PCD is the proposed mechanism by which this was achieved. PCD compromised the survivability of all members of the group by disrupting group-level fitness or releasing harmful metabolites that poison others. Selfish behavior was kept in check under threat of death. In addition to aligning the evolutionary interests of cells in the group, the mediation of conflict by PCD results in further group benefits. The need for the transfer of fitness in evolutionary transitions has been highlighted (Michod, 1999; Bouchard & Huneman, 2013) and cells dying by PCD fulfill this role by releasing nutrients that are absorbed by others in the group (Durand et al., 2016), thus exporting fitness from the individual to the group.

The manipulation of PCD is the basis for social interactions in multiple microbial contexts. In the last two decades, a body of work on diverse taxa identified PCD as a mechanism of social interaction between individuals and their groups (Durand et al., 2019; Michod & Nedelcu, 2003; Vardi et al., 2007; Yordanova et al., 2022). The mechanisms and traits that promote death at the cell level and those that promote survival at the group level exhibit causally related reciprocal changes (Durand, 2021; Koonin & Krupovic, 2019). The implication is that PCD is more than merely a conflict mediator; it is the foundation for a coevolutionary interaction across two levels of social organization—individuals and groups (Durand, 2021). The argument proposed is that the individual-group coevolution of PCD as a conflict mediator and exporter of fitness promoted the formation of social groups and that “a [stable] putative death program evolved from a kind of group selection” (Huneman, 2023, p. 461).

Our organization of what we call the conflict mediation-coevolution hypothesis comprises three elements (1) cell death mechanisms that mediate conflict between individuals, led to (2) the coevolution between death at the individual level and survival at the group level, and ultimately (3) group selection for a stable PCD mechanism.

### Viral defense and the immune hypothesis

Closely related to the immunological concept of PCD is the proposal that PCD arose as a viral defense mechanism. The basic proposition is that unicellular organisms limit the spread of viruses by inducing a PCD pathway before the virus has had a chance to reproduce, lyse the cell, and liberate its progeny. Bacteria have typically been used as model organisms and the molecular mechanisms have, in some cases, been dissected in detail (Fukuyo et al., 2012; Refardt et al., 2013). Crucial to the claim that PCD is a defense mechanism was the finding that (kin) groups with PCD outcompete those without even when relatedness within the populations are low (Refardt et al., 2013).

There is a small, but highly robust, body of experimental (Berngruber et al., 2013; Fukuyo et al., 2012; Refardt et al., 2013) and theoretical/mathematical (Débarre et al., 2012; Vostinar et al., 2019) support for the viral defense-immune hypothesis, which is sometimes more broadly been referred to as a “Suicidal Defense Against Infection” hypothesis (Fukuyo et al., 2012, p. 1). The claim that “bacterial suicide is adaptive and represents a highly efficient host-defence mechanism” (Refardt et al., 2013, p. 5) is the explanatory basis for the potential origin of PCD (Berngruber et al., 2013; Blackstone & Green, 1999; Fukuyo et al., 2012; Koonin & Krupovic, 2019). Interestingly, PCD is not the only outcome of viral infection. Recent experiments reveal that viruses can also induce spore formation as a defense mechanism (Pelusi et al., 2021), and sporulation has previously been included in the PCD spectrum (Durand & Ramsey, 2018). However, there is one caveat worth highlighting. Most of the mathematical models and simulations reveal that population structure is an important element in the evolution of PCD as a defense against pathogens (Débarre et al., 2012; Humphreys & Ruxton, 2019; Vostinar et al., 2019). On the face of it, this requirement does not seem prohibitive. Most prokaryotes indulge in a range of complex social behaviors that lead to the structuring of populations in their native habitats creating groups on which kin/group selection can act (Troselj et al., 2018; West et al., 2007).

## Evaluating the origin of programmed cell death through an evolutionary lens

We previously argued that the evolutionary concept of PCD has important advantages over the others (Durand & Ramsey, 2018). One advantage is that it uses evolutionary histories to distinguish incidental death from PCD, since making this distinction based on mechanisms alone is challenging, if not impossible. In this view, PCD is defined as an adaptation—a trait that has been shaped by its selection history—while other forms of apparently non-incidental cell death not based on adaptations for death are labelled *ersatz*. In this section, we examine the relationship between the evolutionary PCD concept and the five hypotheses for the origin/maintenance of PCD. How does the evolutionary concept line up with the five hypotheses? Does it provide clarity or support for the arguments in their favor?

The evolutionary PCD concept invokes a multilevel selection framework for assigning fitness benefits in the origin/maintenance hypotheses. For PCD to be selected for, an advantage must be accrued at higher levels of biological organization, such as kin groups, populations, or communities (Eronen & Ramsey, In Press). How is this requirement for higher-level selection accommodated by each of the hypotheses? Let’s begin with the multicellularity-cooperation hypothesis, which was developed with a hierarchy of levels in mind.

Since the nineteenth and twentieth century discoveries of PCD—and its forbearer ‘natural cell death’—the assumption was that PCD conferred a fitness advantage to clonal cell groups in multicellular organisms by regulating ontogenesis and tissue homeostasis. The selection of clonal relatives lies at the heart of the multicellularity-cooperation hypothesis. However, multicellularity is an endpoint on a cell cohesion continuum, which ranges from solitary cells to group-structured populations of cells to obligate multicellularity. The multicellularity hypothesis as the only explanation for the origin of PCD seems reasonable only if multicellularity and PCD emerged in tandem. But because of evidence that PCD emerged long before multicellularity, it is not a viable hypothesis for the origin of PCD. Of course, as a mechanism for the maintenance and ongoing evolution of PCD in multicellularity, the hypothesis is justified.

The addiction hypothesis proposes that the origin of PCD occurred with the invasion of the earliest cells by selfish genetic modules (Ameisen, 2002). Cells that purge themselves of the invading plasmid or other autonomous DNA molecules are eliminated by a continuously expressed toxin that outlives the selfish element and the antidote it synthesizes. As discussed in Sect. 3, genic selection is the explanatory framework at play with this hypothesis. However, labeling this kind of cell death, which is caused by gene-level selection, as PCD is inconsistent with the notion that PCD is adaptive for cell groups. This is further illustrated when considering the anthropomorphized language commonly used in the literature. Calling cell death ‘altruistic’ when it is induced by autonomous genes that are uncoupled from the cell’s genome seems misguided. We suggest that in such cases the cells are forced to die by the selfish genes, and that this is distinct from PCD. Cell death of this sort—caused by gene-level selection—can be considered *forced* rather than *programmed* cell death.

In the pleiotropy and original sin hypotheses (POSH), cell death emerged as a by-product of biochemical reactions or a side-effect of pro-survival genes (Ameisen, 2002). The proposal is that PCD originated via the co-option of genes that were involved in metabolic, reproductive, and other cellular functions. PCD was subsequently selected for because of the higher-level fitness benefits accrued by cell groups or at new levels of organization such as eukaryogenesis. The evolutionary concept defines PCD as an adaptation at the level of cell collectives and, therefore, is consistent with the POSH as part of an origins account, since the effects could in the beginning have accidental higher-level benefits. If these benefits were selected for, and if this selection shaped the traits involved in cell death, then the mechanisms underlying the POSH could serve as a foundation for PCD.

Next consider the viral defense-immunity hypothesis. This hypothesis fits well with the origins of PCD (understood in the evolutionary sense), since it involves a multilevel selection framework (the altruistic benefits of cell death are conferred on others). This hypothesis proposes a specific scenario—defense against viral invasion—for the origin of PCD (Koonin & Krupovic, 2019). Conceptually, the hypothesis provides a highly plausible account for the origin of PCD. Empirically, comparative genomic analyses reveal that host-virus interactions were ubiquitous at the origin of life and played a central role in shaping cellular genomes (Moelling, 2012). Most, if not all, extant cells remain vulnerable to viral-induced death, and it seems reasonable to assume that the survival of cells at the origin of life depended on the evolution of a defense mechanism that prevented viruses from sweeping through whole populations of cells. As discussed in Sects. 2 and 3 above, even a relatively rudimentary genetically encoded cell suicide mechanism in bacteria provides robust immunity against viruses at the level of populations (Berngruber et al., 2013; Refardt et al., 2013; Fukuyo et al., 2012). Furthermore, the viral defense-immunity hypothesis posits that PCD is an adaptive cell response, which is consistent with the evolution-based conceptualization of PCD as a group/kin level adaptation.

The conflict mediation-coevolution hypothesis proposes a potentially viable alternative to the immunity-viral defense hypothesis. Our analysis, however, suggests that conflict mediation and coevolution are more plausible as maintenance mechanisms than as mechanisms for the origin of PCD. The primary reason is that the hypothesis rests on the existence of relatively well-defined groups. Conflict mediation implies the emergence of cell groups, and cells and cell groups are both required for the coevolutionary dynamic between traits at the individual and group levels. In contrast, the viral defense-immunity hypothesis has no such requirement and PCD is highly effective at limiting viral spread in diffuse cell populations without the need for group structures. Thus, we argue that the viral defense-immunity hypothesis is a more parsimonious account, backed up by mathematical simulations and empirical data.

In considering the possible role of viruses in the origins of PCD, we can explore whether and how PCD could have evolved in a world without viruses. Because viruses use cells to replicate, it is sometimes argued that they arose after the evolution of the first cells (for a review of viral origins see Forterre, 2006). Comparative genomic data reveal that at least some pro-death genes trace back to the earliest cells. As some researchers suggest, these may be pleiotropic and “supports the original sin hypothesis for the origin of PCD” (La et al., 2022). In this case, it would not have been true PCD in the beginning, though if the pleiotropic effects carried a higher-level benefit, subsequent selection could have transformed it into PCD.

A more straightforward path to PCD in a virus-free world would be via conflict mediation, coevolution, and group selection. The argument is that, in the absence of viruses, PCD was a mechanism for surviving the extreme nutrient-limited environments typical of the early Earth. The argument is supported by mathematical models (for example, Tanouchi et al., 2012) and cells survived because of the social exchanges brought about by recycling nutrients, trace minerals, and other resources liberated by dying cells and available for uptake by others. Such an account is consistent with recent fossil evidence (Jodder et al., In Press). However, it is currently unexplored and exists only as a speculative explanation for the origin of PCD in a virus-free world.

## Concluding remarks

In this article we organized the concepts and the origin/maintenance hypotheses of PCD into coherent definitions and arguments, tracing the history of conceptualizing cell death and arguing for how PCD is best conceptualized in light of current evidence. We argue for the strengths of the evolutionary concept of PCD and hold that it sharply distinguishes PCD from other forms of endogenous cell death and clarifies the existing hypotheses for the origin and maintenance of PCD. Our analysis suggests that the viral defense-immunity hypothesis offers the most plausible account for the origin of PCD. Furthermore, the account is consistent with the current empirical and comparative genomic data, as well as mathematical models and simulations. We suggest that the conflict mediation-coevolution and multicellularity-cooperation hypotheses are viable as proximate causes for the maintenance and ongoing evolution of cell death in a range of sociobiological contexts that emerged following the origin of PCD.
